# The Use of Microfabrication Techniques for the Design and Manufacture of Artificial Stem Cell Microenvironments for Tissue Regeneration

**DOI:** 10.3390/bioengineering8050050

**Published:** 2021-04-23

**Authors:** David H. Ramos-Rodriguez, Sheila MacNeil, Frederik Claeyssens, Ilida Ortega Asencio

**Affiliations:** 1 Bioengineering and Health Technologies Group, The School of Clinical Dentistry, University of Sheffield, Sheffield S10 2TA, UK; dhramosrodriguez1@sheffield.ac.uk; 2 Biomaterials and Tissue Engineering Group, Department of Materials Science and Engineering, Kroto Research Institute, University of Sheffield, Sheffield S3 7HQ, UK; s.macneil@sheffield.ac.uk (S.M.); f.claeyssens@sheffield.ac.uk (F.C.)

**Keywords:** microfabrication, microtopographies, stem cell microenvironment, tissue regeneration

## Abstract

The recapitulation of the stem cell microenvironment is an emerging area of research that has grown significantly in the last 10 to 15 years. Being able to understand the underlying mechanisms that relate stem cell behavior to the physical environment in which stem cells reside is currently a challenge that many groups are trying to unravel. Several approaches have attempted to mimic the biological components that constitute the native stem cell niche, however, this is a very intricate environment and, although promising advances have been made recently, it becomes clear that new strategies need to be explored to ensure a better understanding of the stem cell niche behavior. The second strand in stem cell niche research focuses on the use of manufacturing techniques to build simple but functional models; these models aim to mimic the physical features of the niche environment which have also been demonstrated to play a big role in directing cell responses. This second strand has involved a more engineering approach in which a wide set of microfabrication techniques have been explored in detail. This review aims to summarize the use of these microfabrication techniques and how they have approached the challenge of mimicking the native stem cell niche.

## 1. Introduction

Stem cells are ideal candidates to be used in regenerative medicine applications due to their perpetual self-renewal capabilities and their ability to differentiate into specific tissue types. The three main categories of stem cells (according to their origin) are embryonic, fetal, and adult stem cells. There are significant ethical burdens associated with the use of embryonic or fetal stem cells which are related to medical regulations and consents as well as clinical challenges associated with the control of stem cell fate [1,2]. Adult stem cells, however, overcome some of these challenges since they present limited capacity to differentiate, and they are present in mature tissues and can be accessed with full and informed consent from the donors.

To fully understand and control adult stem cell behavior it is imperative to also study the spatial, mechanical, and topographical cues that are present in the adult stem cell native microenvironment (stem cell niche) [3,4]. 

Over the last decades, the introduction of micro and nano topographies within tissue engineering constructs (for example microgrooves, nanopits, and micropillars) has demonstrated a significant effect in guiding and controlling cell behavior; it has been reported that changes in cytoskeletal distribution and alignment of the cells have been proved to modulate several aspects of cell function and tissue regeneration, including protein production, cell fate, and proliferation [5,6].

Understanding and controlling the stem cell microenvironment opens the door to a broad new range of applications in the healthcare sector not only based on the possibility of creating innovative analytical tools and drug screening models, but in the field of regenerative medicine. With the development of novel biomaterials and manufacturing techniques, tissue engineers have now the tools to create new devices that can provide structural, chemical, and biological cues similar and mimic closer to the stem cell native environment. 

In order to address the difficult and challenging task of mimicking the complexity of the stem cell niche, scientists have followed different strategies that can be divided into two main groups: (1) Mimicking metabolic behavior and biological interactions and (2) mimicking micro-nano architecture and spatial/geometrical control. This review is focused on describing the different manufacturing and microfabrication techniques used to date for the development of stem cell niche microenvironments by mimicking its spatial organization. However, a brief summary of efforts focusing on mimicking the molecular environment (1) is also included. Before discussing how microfabrication techniques have contributed to the development of synthetic niche-like environments it is important to define the stem cell niche and identify why it is important to introduce the stem cell niche concept within biomaterial design.

## 2. The Adult Stem Cell Microenvironment

Adult stem cells are considered multipotent cells as they only differentiate to cell types related to the tissue in which they reside. Types of adult stem cells include neural, hematopoietic, mesenchymal, and epidermal. After birth, somatic, germline, and adult stem cell populations reside in defined microenvironments called stem cell niches, which provide protection and use physical proximity and extracellular cues to regulate maintenance and function [7,8]. 

**Figure 1:**
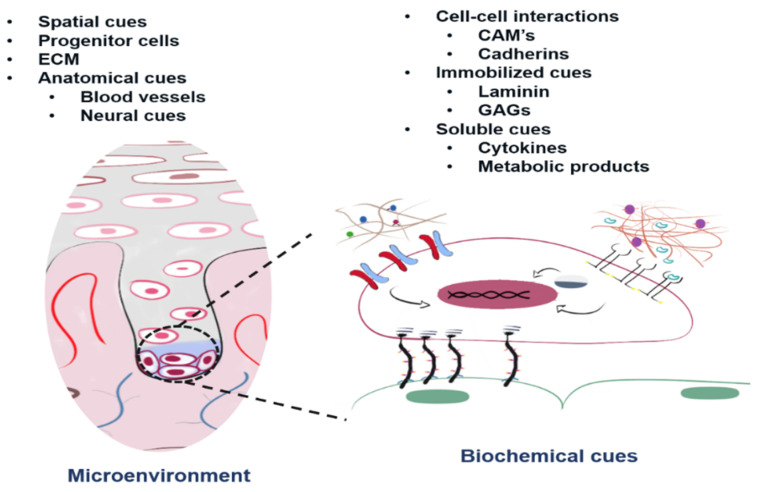
Biochemical and microenvironmental cues of a prototypical stem cell niche. Adapted with permission from ref. [9]. Copyright Lutolf & Blau’s Name.

The components of a prototype niche can be grouped in structural (physical) or biochemical (Figure 1). The 3D environment of a stem cell niche is believed to (i) provide a well-defined space that allows a certain degree of physical protection, (ii) supply distinctive and crucial extracellular matrix (ECM) components to accommodate a cell population with stem cell capacity and (iii) allow the presence of stromal cells and specific soluble factors [9,10].

The existence of the cell niche as a physiological microenvironment that supports stem cells was proposed and defined in 1978 [11]. However, the relation between the structure of the niche and how it affects stem cell behavior was first described two decades later by using genetic model systems such as *Caenorhabditis elegans* [12] and *Drosophila* [13,14]. 

**Figure 2:**
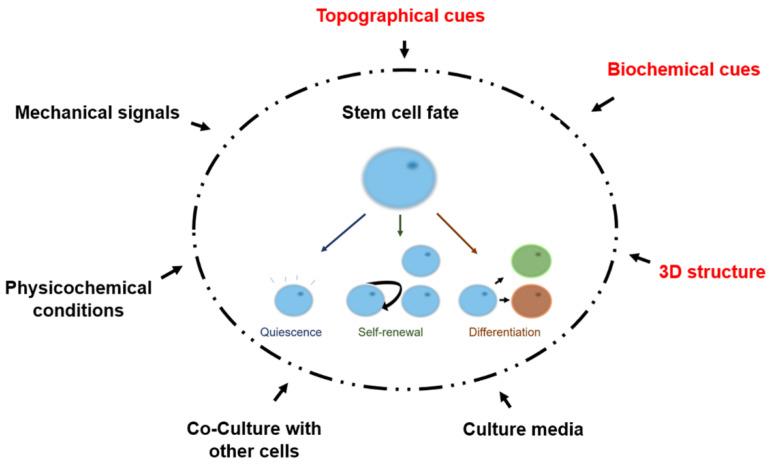
Schematic representation of environmental factors that affect stem cell fate. The factors highlighted in red are discussed in this review. Mechanical signals although intrinsically related to spatial distribution and topography are not covered in this review. Adapted with permission from ref. [15]. Copyright 2003. Copyright Jiang and Papoutsakis’s Name.

The structural and biochemical cues present in the stem cell niche provide key signals that control stem cell fate and protect stem cells from death or exhaustion. The microenvironment created by the niche encourages a series of close-range signals such as pH, ECM components, and their binding to integrin transmembrane proteins, and soluble proteins. These niche signals are tissue specific and require a specific spatial distribution to maintain their function. The niche can also react to mechanical and metabolic stimuli to induce specific stem cell function [15]. The cellular organization is critical to the function of the niche, as autocrine and paracrine signals from the stem cell population and cell-cell contact influence stem cell activity [16]. Figure 2 shows the environmental factors that can influence stem cell fate.

Several studies have shown the impact of aged and deregulated stem cell niches in vivo, suggesting a relation between the loss or disruption of the stem cell niche (and the systemic factors expressed by the niche) with pathologies associated with tissue regeneration, premature aging, and tumorigenesis [17,18,19]. Moreover, the organization of the stem cell niche has been proposed to protect the stem cells from the accumulation of gene mutations that may lead to malignant transformation [20].

The low accessibility of the stem cell niches has limited their study in humans, where the location and regulation mechanism of most stem cell niches remains elusive. Nonetheless, stem cell niches located in the bone marrow, hair follicles in the skin, and the small intestine are particularly interesting for their role in stem cell fate and tissue regeneration. Table 1 summarizes the tissues, their niches, and stem cell populations that have been studied for their importance to regulate tissue regeneration.

Changes in composition or distribution could disrupt the balance of cell renewal and compromise the lifespan of the tissue. Systems that require a high proliferation and continuous replenishment of cells, such as the epidermis, intestine, cornea, or hematopoietic system, have a need for active stem cell niches that can supply this high self-renewal cycle [38,39,40,41].

In comparison, tissues such as the skeletal muscle or the endoskeletal region require a dormant stem cell niche that can preserve a quiescent stem cell population [17,18,19,20,21,22,23,24,25,26,27,28,29,30,31,32,33,34,35,36,37,38,39,40,41,42]. This behavior can be observed in epithelial stem cells where the proliferative potential, physical protection, and undifferentiated phenotype are defined by the microenvironments present on the corneal limbus, hair bulge, epidermal layer, or at the bottom of the rete ridges [21].

Although micro and nano topographical cues have been introduced within biomaterial constructs to provide substrates to mimic cell-cell and cell-niche interactions, the reality is that recreating the stem cell niche microenvironment remains a big challenge [5,6,7,8,9,10,11,12,13,14,15,16,17,18,19,20,21,22,23,24,25,26,27,28,29,30,31,32,33,34,35,36,37,38,39,40,41,42,43] and the currently available tissue engineering scaffolds fail to recreate the native tissues either at micro or nanostructural levels.

As highlighted in the introductory section, the main strategies reported in the literature that aim to recreate stem cell niches can be grouped into two distinct categories: (a) introducing complexity into tissue engineered scaffolds by mimicking to a certain extent the natural spatial/physical microfeatures and (b) mimicking the complex biological components that influence cellular metabolic behavior and molecular interactions.

## 3. Mimicking Biological Components of the Stem Cell Microenvironment

The study of the molecular components of the stem cell niche has been critical to understanding the mechanisms involved in stem cell regulation. Studies with *Drosophila* have shown an intrinsic relationship between acellular components produced in the niche and stem cell homeostasis [44,45].

The initial attempts to recreate to a certain extent the stem cell niche started with deconstructing the biochemical cues of the niche. Introduction of ligands and peptides, changes in matrix composition and mechanical properties, co-culture with stromal support cells, and addition of soluble factors (chemokines, cytokines, and growth factors) have been some of the strategies used to mimic the complexity of the stem cell niche [17,46].

The ECM of the stem cell niche has been the main focus to replicate due to its importance in controlling cell behavior through cell receptors, signaling molecules, structural proteins, glycoproteins [47], enzymatic remodeling, metabolic products, and growth factors [48]. Cells interact with the ECM to form adhesion to the nanometric fibers that mostly constitute the extracellular topographical environment [49]. These interactions are controlled by integrins, transmembrane proteins, and their binding to specific amino acid sequences such as the RGD sequence found in fibronectin [50,51].

The role of integrin and non-integrin receptors have been explored due to their role in homing, adhesion, anchoring, proliferation, differentiation, survival, and migration on stem cell microenvironments [52,53,54,55].

However, most of these receptors have yet to be introduced into scaffolds. Early attempts at mimicking a functional hematopoietic stem cell niche were published by Sharma et al. in 2012, using co-cultured mesenchymal stromal cells and CD34+ cells introduced into hydrogels to influence stem cell phenotype and functional parameters [56]. Comprehensive reviews of the methodologies used to study the biological components of the adult stem cell niche were presented by Lutolf et al. [9,57].

Biomolecular patterning is a promising approach to create gradients of biochemical cues on surfaces that can later be introduced into spatial cues to create a complex microenvironment. Protein microarrays, on-chip microdroplets, and biomolecular patterning are recent platform technologies that are being used to introduce molecular complexity within scaffolds and to study how stem cells behave in vitro [57].

## 4. Mimicking the Spatial and Physical Components of the Stem Cell Microenvironment

Using microfabrication techniques such as additive manufacturing and soft-lithography scientists have been able to create complex arrays and architectures that were not possible before. Microfabrication techniques involve the construction of structures, patterns, or topographies that range in the micrometric scale (or nanometric for several applications). The first attempts at controlling and understanding the relation of spatial distribution and cell behavior started with the introduction of fibrous environments to mimic the ECM [58,59].

Initial studies have proved that micro-interactions and nanotopography can affect stem cell mechanotransduction through focal adhesion interactions [60], as well as introducing spatial cues that change the distribution of stem cells that regulate stem cell division.

A key factor in selecting a microfabrication technique is considering the nature of the material to be used since chemical functionality, degradation rate/by-products, surface energy, nanotopography, and stiffness, can induce or inhibit specific pathways that control stem cell fate [61,62].

Fabrication techniques that have been used to replicate spatial cues of stem cell microenvironment include soft-lithography (using electron-beam and photolithography), patterned hydrogels, laser-based methods, and electrospinning.

### 4.1. Soft-Lithographic Methods

**Figure 3:**
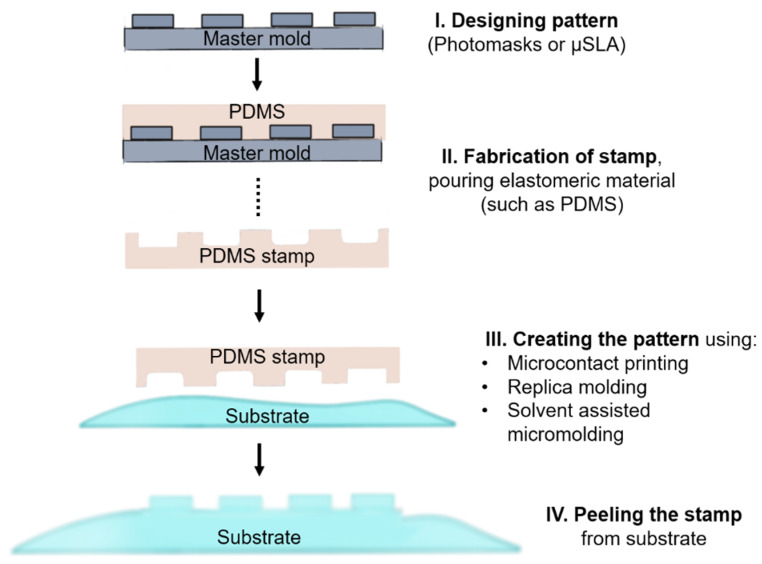
Soft lithography fabrication diagram. First, a master mold is created normally using a silicon wafer. The CAD pattern is printed on the surface of the master mold using µSLA. Once completed, the elastomeric material is poured, and a stamp is created. This stamp is used to create a pattern on the substrate surface. The patterning technique depends on the selected substrate, which is chosen according to the application. Finally, the stamp is peeled from the substrate.

Soft lithography is a family of fabrication techniques that uses elastomeric stamps, molds, and conformable photomasks, that range from micrometer to nanometer scale to create patterned surfaces [63]. The stamp is generally prepared by casting the liquid pre-polymer of an elastomer material against a master material that has been created via traditional lithographic techniques such as photolithography or stereolithography (SLA) [64] and thus is limited by the resolution of the method chosen to create the stamp. In some of these studies, µSLA is used which refers to building high resolution (100 nm–10 µm) structures with stereolithography and related techniques such as 2PP. Controlling the peeling rate and selecting a proper patterning technique is critical to avoid deforming the stamp or crushing the printed pattern [65,66]. Micro-contact printing [67], replica molding [68], solvent-assisted micromolding [69], and micromolding in capillary [70] are stamping techniques that have been used to preserve micro topographical cues for cell culture. Figure 3 shows the general fabrication method for soft lithography patterned surfaces.

Advanced photolithographic techniques have been raised to overcome the resolution limitation. Electron-beam lithography (eBL) uses a narrow beam of electrons over an electron-sensitive material allowing for a lateral high resolution of 3–5 nm [71], higher than the 1 µm resolution of standard photolithography The high resolution of eBL has been used to study populations of undifferentiated MSCs seeded on nanotopograhical cues from 120–500 nm [72,73,74].

The polymer PDMS is the standard material to create the stamp due to its elasticity, hydrophobicity, biocompatibility, and optical transparency [75]. Nonetheless, it is possible to transfer the pattern using other elastomeric materials such as polyurethane, polyamide, phenol-formaldehyde polymers, and specific siloxane polymer formulations for high-resolution applications [76,77]. Synthetic and non-synthetic biomaterials can be used in this technique to create micro or nanostructures to study cell behavior. Materials such as poly(ethylene glycol) (PEG) [78], poly(lactic-co-glycolic acid) (PLGA) [79,80], collagen [81], and elastin are commonly used to either functionalize the PDMS stamp or as a casting material. Although soft-lithographic stamps can be used directly for cell culture, the functionalization of the stamp is required to achieve appropriate cell adhesion.

The most common approach for soft lithography in tissue engineering is casting the PDMS pattern on a polymer with surface properties more suitable for cell culture or that possesses specific biochemical and mechanical properties for the desired application. It is also possible to load the PDMS stamp with the relevant biomolecules that are then printed on the surface of the cast polymer [82], creating a “hybrid” scaffold that combines biomolecular patterning and spatial cues to mimic the stem cell microenvironment.

Table 2 shows some of the work that has been done to replicate and study the stem cell microenvironment using soft lithography. The initial attempts of introducing topographical cues to stem cell cultures were done by using patterned surfaces created by lithographic techniques without further functionalization [83,84]. Further developments in stem cell research showed the importance of introducing substrates that encourage rapid stem cell attachment to avoid undesired differentiation [85,86,87]. This led to the use of stiff biocompatible substrates that can later be functionalized with surface modification techniques or by a bioactive coating [88].

Soft lithography has been widely used due to the variety of available materials, versatility when designing the micropattern, and relatively low cost [65,98]. However, generating angled surfaces, and producing densely arrayed patterns for nanometric applications remain challenges for conventional soft lithography [68,99]. In vivo applications of this fabrication technique are limited. This can be related to the poor biodegradable and/or biocompatible characteristics of the most common substrate materials.

It is important to consider that the extracellular fibrous environment in the stem cell niche is key for nutrient diffusion, extracellular signals, and cell migration. However, most soft-lithography applications recreate the micro or nano spatial cues on solid 2D substrates. This means that the constructs created with this technique have low to no cell infiltration potential and cannot replicate the 3D fibrous environment of the ECM or the porous structure of hard tissues. To improve cell infiltration and thus tissue integration and construct survivability, collagen has been incorporated into soft-lithographic applications either by coating the elastomeric mold or as the cast material [94,100].

Although it is possible to use a conventional PDMS stamp on a hydrogel substrate, the physical properties of the hydrogel require different considerations from those that a solid substrate to preserve the microstructure of the pattern. In recent years, advanced soft-lithographic micropattern techniques have been developed to create high-resolution and high-throughput microfluidic scaffolds and patterned hydrogels.

#### 4.1.1. Patterned Hydrogels

Hydrogels are defined by their 3D polymeric environment and their hydrophilic nature capable of holding large amounts of water. Their high biocompatibility and versatile biodegradability have made hydrogels excellent biomaterials to be used as drug delivery devices, hemostasis bandages, biosensors, and carriers for cells in tissue engineering [101]. Although hydrogels can be fabricated from synthetic and natural polymers, synthetic polymers have become more relevant due to their long service life, high mechanical strength, and higher water absorption [102]. Relevant hydrogel materials used to culture stem cells include PEG [103], poly(2-hydroxyethyl methacrylate) (HEMA) [104] elastin [105], collagen [106], and hyaluronic acid [107].

Hydrogels are commonly fabricated by copolymerization or crosslinking free-radical polymerizations inducing hydrophilic monomers to react and form a network [102]. Their 3D spatial network of polymeric chains made hydrogels a promising alternative to recreate the stem cell microenvironment ECM. Because the cross-linking density alters the stiffness of the hydrogel, it is possible to study the relation between the mechanical properties of the substrate and stem cell behavior [108,109].

Collagen hydrogels are examples of natural polymers used to recreate the ECM’s physical and chemical components [110,111]. Other natural materials used to fabricate hydrogels include fibrin, gelatin, alginate, and hyaluronic acid. Hydrogels made with these natural materials can promote cell adhesion due to the presence of bioactive sequences that interact with protein receptors [112]. Bioactive modifications of synthetic hydrogels can enhance cell attachment and proliferation by introducing Arg-Gly-Asp (RGD) peptide sequences, growth factors, ECM-derived short peptides, and proteoglycans [113].

Conventional hydrogels introduce a nanometric 3D network but lack the microarchitecture present in the stem cell niche. The need to recreate both the 3D environment and topographical cues lead to the development of several fabrication methods, aiming to introduce micro/nano spatial cues without compromising the chemistry and mechanical properties of the hydrogel. These techniques include: glass slide nanopatterning by block-copolymer micelle nanolithography (BCMN) [114,115], multilayer soft-lithography [116], Real Architecture For 3D Tissue (RAFT^TM^) [117,118], and capillary force lithography (CFL) [119]. Table 3 shows examples of patterned hydrogels fabricated to replicate the stem cell microenvironment.

In comparison with conventional soft-lithographic methods, fabricating patterned hydrogels is a longer and more complex procedure, especially for biofunctionalized hydrogels. Although it is possible to use hydrogels as a substrate for soft-lithography, soft hydrogels are usually damaged by conventional demolding or curing steps [123]. However, a microstructured hydrogel construct is arguably more efficient in recreating the stem cell microenvironment spatial cues as it incorporates a 3D nanometric network as well as a 2D patterned surface that can mimic specific niche architectures. Because of their high biocompatibility, hydrogels are commonly implemented for in vivo applications to reintroduce stem niche-like structures on damaged tissue [119,120].

Stimuli-responsive hydrogels are yet to be tested as a platform to introduce micro topographical cues and study the components and mechanism of stem cell niches. However, they are a promising approach to study adult stem cells in a dynamic physical and chemical microenvironment [124,125].

#### 4.1.2. Microfluidic Devices

Microfluidic systems are based on the control of fluids in a micrometer to millimeter scale, allowing for precise control of soluble factors, gradients, and mechanical signals in which biological systems can be studied [126]. Microfluidic scaffolds are a promising strategy to recreate and manipulate the 3D structure of the stem cell microenvironment while dictating the distribution and flow rate of soluble biomolecules [127]. Microfluidic techniques had led to the development of high throughput cell culture systems and organ-on-a-chip devices [128].

Microfluidic devices make an excellent platform to study cells under various physical, chemical, and mechanical microenvironmental conditions such as stress capillary flow, chemical gradients, pH, temperature, micro, and nano spatial cues, and the effects of single/low cell numbers on the temporal and spatial resolution [129,130,131].

Applications of microfluidic techniques were primarily used to study single-cell interactions in a controlled microenvironment that can recreate certain biologically relevant conditions. Although there is no standard procedure to fabricate a microfluidic device, common fabrication techniques include droplet-based microfluidics, micro-molding, and sacrificial layer elimination [132,133,134,135]. Microfluidic fabrication techniques have been covered extensively in other reviews [127,134,136]. Table 4 shows a summary of microfluidic devices fabricated to replicate certain aspects of the stem cell microenvironment.

Although versatile in their applications, microfluidic devices required a complex setup and long fabrication process. Regarding stem cell culture, major limitations for long term applications include liquid evaporation, leaching of non-reactive compounds, hydrophobic recovery, and protein adsorption [129]. Microfluidics is a powerful tool to understand the underlying mechanism of the stem cell microenvironment, as it allows for the measure and control of relevant metabolites and the platform can be as complex as needed. Novel applications have aimed to introduce several layers of parallel reactions to mimic several adjacent mechanisms that control cell fate.

### 4.2. Electrospinning-Based Methods

**Figure 4:**
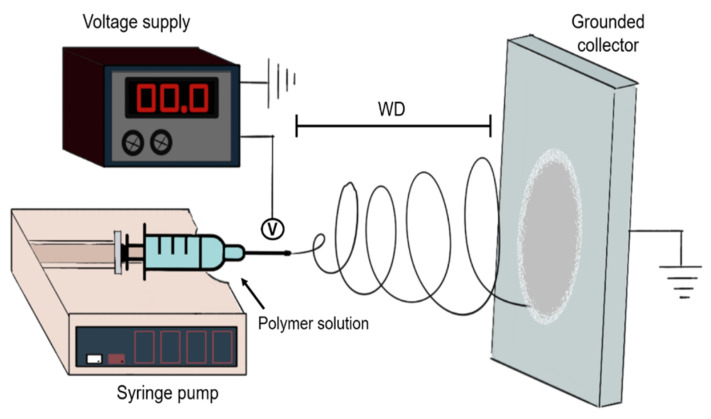
Diagram of the fabrication of nano/microfibers using a conventional electrospinning setup. First, a polymer solution is a pump through a capillary, commonly using a syringe pump. Then the working distance (WD) is set. The high voltage source with positive polarity is connected to the capillary and negative polarity connected to a grounded collector. As the polymer solution flows through the tip of the capillary it forms a jet that travels towards the collector.

Electrospinning is an advanced manufacturing technique that uses polymer solutions and an electrical potential to produce fibers from nanometer to micrometer scales. Electrospinning techniques have the potential to control fiber diameter and orientation in comparison with other techniques such as hydrogels which can be fabricated from fibrous components, but which distribution is intrinsic to the material. In comparison, electrospinning uses different setups, and changes in the solution and process parameters to generate specific micro and nanofibrous architectures [143]. The standard electrospinning setup is represented in Figure 4.

Electrospinning fibers are fabricated by electrifying a pendant drop of polymer solution until the electrostatic charges disrupt its surface, deforming the drop and creating a conical shape known as the Taylor cone. If the voltage continues to increase over the threshold value of the polymer solution, the surface tension of the Taylor cone is disrupted creating a polymer jet that travels toward the grounded metal collector. As the polymer jet travels, the solvent evaporates and with a constant polymer flow rate, a nonwoven fibrous mat is deposited on the collector [144,145].

The process parameters (voltage, the distance between the drop and the collector, and flow rate), solution parameters (surface tension, solvent system, viscosity, solution conductivity), and environmental parameters (temperature and humidity) control the outcome of electrospinning. The general relation between these parameters has been described previously [145], but no model has been able to predict the dynamic electrospinning phenomena [143]. Meaning that all new applications should be thoroughly tested, and parameters should be refined for each application.

The relation between fiber diameter and cell migration and adhesion has been well established for primary cell lines [146,147]. The high porosity and structural properties of nanofibers have also been proved to enhance cell differentiation, adhesion, and proliferation. Synthetic and natural materials can be processed to create electrospun mats. These materials include PLGA, polylactic acid (PLA), poly(e-caprolactone) (PCL), PU, collagen, elastin, and silk [148,149,150]. However, for most natural polymers their low solubility, high molecular weight, and denaturation issues within the solvent system (especially for collagen electrospun fibers), have limited their applications [151,152,153,154]. Combinations such as PLGA-collagen/gelatin [155,156], PCL-elastin [157], and PLA-silk [158], have reported that biocomposite fibrous scaffolds enhance cell adhesion and proliferation while providing suitable mechanical properties.

The flexibility of electrospinning has made it a promising approach for several biomedical applications such as dressings, tissue engineering scaffolds, and drug delivery devices. Table 5 shows examples of studies that have used electrospun fibers to study how stem cells interact with their fibrous ECM environment and how they respond to topographical cues (aligned or random fibers).

Although most studies are based on the capacity of electrospun mats to resemble ECM nanofibrous nature, conventional electrospinning approaches have limitations regarding their capacity to recreate both nano and micro topographical components in one construct. As electrospinning excels at creating nanofibers up to 9 nm [163], but the process became unstable as fiber diameter increases above 10 µm [164]. However, recent studies conducted by Ortega et al., have shown the potential of electrospinning to create microenvironments by combining conventional electrospinning with selective laser melting (SLM) [165] and µSLA [166,167]. These approaches are covered in detail further in this review.

### 4.3. Other Fabrication Methods

Increasing interest in replicating stem cell microenvironment has led to the use of novel techniques such as melt spinning or bioprinting, and to the refinement of processes to improve currently established techniques such as the use of two-photon polymerization (2PP). These techniques, although relevant to introduce stem cell niche-like features, will not be covered extensively in this review.

Melt spinning is an advanced manufacturing technique in which the polymer is extruded through a spinneret in a molten form, without the use of solvents. Two polymers can be coextruded to create a single filament with a designed cross-sectional arrangement. This technique is normally used in tissue engineering to generate macrofibers and to better control fiber alignment and pore size [168,169]. For example, melt spinning PLA fibers about 10-20 µm have been fabricated and polymerized with polypyrrole to study the effects of electrical stimulation on osteogenic differentiation of hASCs [170].

Bioprinting is the combination of 3D printing technologies (such as extrusion, inkjet, or laser-assisted) using cell and biocompatible supporting materials to create sections or entire artificial organs for regenerative medicine applications [171]. Current bioprinting methods mainly use extrusion based printing of cells, tissue spheroids, and biomaterials to achieve more complex and functional 3D structures [172]. A clear advantage of bio-printing is that it is possible to combine multiple bioinks (such as collagen, alginate, PEG, and PCL), cell types, and materials into complex 3D structures to mimic highly specific cell-matrix interactions within the stem cell microenvironment [173]. An example of this fabrication technique is the use of thermal inkjet with hMSCs suspended in PEGDMA co-printed with ceramic [174]. Other applications of adult stem cell 3D bioprinting are covered in detail by Ong et al. [175].

2-photon-polymerisation is a light-based manufacturing technology that uses short pulse length (femtosecond) lasers to polymerize photocurable resins via 2-photon absorption. This advanced fabrication technology can create 3D structures with a spatial resolution down to 100 nm. It requires specific photopolymers that are usually modified ceramics, acrylic monomers, or polymerizable epoxides such as SU-8 [176]. The most common approach of using 2PP to recreate the stem cell microenvironment architecture is in combination with other techniques, such as creating precise molds for soft-lithography or hydrogel patterning. Nonetheless, several authors have reported its use more directly, by creating 3D devices that aim to replicate stem cell niches using photocurable biocompatible polymers [177] or hybrid organic-inorganic materials [178].

### 4.4. Combining Micro and Nanofabrication Techniques

As fabrication techniques become more available, the combination of one or more techniques has become feasible for research laboratories. The idea of overcoming certain limitations of a technique by integrating another fabrication method has proved successful for different applications. Table 6 summarizes attempts to introduce elements of the stem cell microenvironment by combining two or more manufacturing techniques. The low-cost setup and available polymers have made electrospinning a common technique that can be introduced in established or new constructs. In general, fabrication techniques are combined when a standalone process cannot provide either mechanical, chemical, physical, or spatial cues for the researchers to study stem cell fate.

## 5. Future Perspective and Concluding Remarks

The role of adult stem cells in the regenerative cycle of human tissues, their ability to differentiate into cells of the tissue from which they derive, and their relatively simple isolation protocols make them a promising tool to be used in regenerative medicine strategies. However, since their discovery, in vitro expansion of adult stem cells has remained a challenge due to the lack of native spatial and biochemical cues on traditional 2D culture platforms. Introducing complex physical cues within in vitro models can be a powerful approach to develop new routes for the study and control of stem cell behavior.

Soft-lithographic methods are easy, non-expensive techniques to recreate 2D surfaces with nano and micro features, however, they fail to provide a 3D network structure and have limited in vivo applications due to the chemical nature of the fabrication method. Complex fabrication techniques such as patterned hydrogels and microfluidic devices were initially developed using the basic principles of soft-lithography and therefore share certain techniques and challenges.

Hydrogels can introduce a 3D structure and can be patterned to create topographical cues that resemble stem cell niches. They can be fabricated from natural and synthetic materials and have been used for several in vivo applications due to their high biocompatibility, especially for natural materials. However, natural hydrogels such as alginate or collagen gels exhibit poor mechanical properties that usually require a post-processing covalent crosslinking step for most tissue engineering applications. This crosslinking step can compromise their biocompatibility due to the toxicity of the degradation products or reduced their bioactivity due to side-chain modifications [184,185,186]. Moreover, synthetic hydrogels need to be functionalized to further improve cell adhesion.

Microfluidic devices are mainly used as in vitro models rather than being a platform for tissue engineering applications. However, they excel at controlling and mimicking all components of the stem cell niche as a functional unit and they have become a gold standard for the development of in vitro studies of single and small populations of stem cells. The main limitation of microfluidic devices is their complex multi-step process, generally requiring different types of equipment as well as exhaustive control of multiple process parameters.

Electrospinning is used on the basis that it can replicate to a close degree the 3D fibrous environment of the stem cell niche [46,187,188,189]. Although it is mostly used for creating aligned or random fibrous environments, controlling fiber diameter and porosity can be achieved with simple modifications to the process or solution parameters. Its versatility to produce nano and microfibers from both natural and synthetic polymers has expanded the use of electrospinning in recent years. Moreover, electrospinning is an easy to perform and scalable process that has been used in several in vivo and in vitro tissue engineering applications, but on its own, it has limited applications for complex constructs to mimic the stem cell microenvironment.

Combining microfabrication techniques may lead to multifunctional complex tissue engineered constructs that could replicate several components of the stem cell niche and overcome individual challenges of each technique. Electrospinning seems to be the most versatile technique and can be successfully combined with other fabrication methodologies including soft lithography and micro-stereolithography. Although all the techniques reviewed here can also be further enhanced by introducing relevant biomolecules, microfluidic devices remain the most suitable in vitro model to test new biochemical cues and their effects on cell behavior as they allow for gradient concentration and controlled metabolite quantification.

All the methods described here introduce complexity to conventional 2D cell culture, and except for conventional electrospinning, their resolution depends directly on the photolithographic method used to create the master mold and the patterning technique. Introducing novel high-resolution techniques such as two-photon lithography to the fabrication of the master mold would significantly improve the spatial resolution of current lithographic constructs [190].

In summary, for achieving the successful incorporation of artificial microenvironments within future tissue engineering scaffolds for each particular tissue, the identification of the appropriate fabrication technique would be critical since it will ultimately define the potential complexity of the construct and therefore its functionality. Combining microfabrication techniques with innovative biofunctionalization strategies would certainly facilitate the creation of dynamic 3D environments where stem cells would be able to reside and differentiate on demand, leading to promising therapeutic approaches for tissue regeneration.

## Figures and Tables

**Table 1:** Stem cell niches which have been studied for their potential to improve tissue regeneration by controlling stem cell behavior.

**Tissue/System**	**Location**	**Stem Cell Population**	**Ref**
Skin	Hair follicles	Melanocyte stem cells, Hair follicle dermal papilla cells	[21,22]
	Rete ridges	Epidermal stem cells	[23]
		Keratinocyte stem cells	[24]
Hematopoietic system	Bone marrow	Hematopoietic stem cells (HSCs)	[25]
		Mesenchymal stem cells (MSC)	[26]
Small intestine	Epithelium of the small intestine	Intestinal stem cells, non-epithelial stromal cells, myofibroblasts.	[27]
Heart	Epicardial lining	Cardiac stem cells (CSC)	[28]
	Myocardium		
Cartilage	Articular cartilage	Bone marrow Mesenchymal stem cells (BMSC), Cartilage-derived mesenchymal progenitors	[29]
Eye	Corneal limbus/Palisades of Vogt	Limbal epithelial stem cells	[30]
Neural system	Subventricular zone	Neuronal stem cells (NSC)	[31,32]
	Hilus of the dentate gyrus	Radial neural stem cells, Dentate gyrus neural stem cells	[33]
Lung	Lung epithelium/tracheal submucosal glands	Basal cells, club cells, and alveolar epithelial cells type II cells.	[34,35]
Primary or permanent teeth	Dental pulp tissue	Human dental pulp stem cells, MSCs, BMSCs.	[36,37]

**Table 2:** Summary of soft-lithographic constructs used to recreate the micro and nano spatial cues of the stem cell niche.

Application	Polymer	Outcome	Ref
Study MSC fate and neurosphere formation.	PDMS mold cast on PEG hydrogel.	96-well plate structure. Each well is composed of 33 × 33 microwells of 100 µm diameter.	[78]
Observe retinal progenitor cell behavior.	PDMS mold cast on PLGA 75:25 substrate.	Microchannels of 15 µm diameter and 40 µm height.	[80]
Assess the effects of ridges and grooves on hMSCs differentiation and proliferation.	PDMS stamp cast on NOA81 polyurethane	Microgrooves of 300 nm in depth and 400, 1400, or 4000 nm pitch.	[89]
Study keratinocyte stem cell niches of the dermal-epidermal junction	Collagen type I pour on PDMS mold. Collagen was then conjugated with fibronectin	Multilayer constructs with a series of 200 µm deep channels with variable widths of 50, 100, 200, and 400 µm.	[90]
Analyze the response of hHSC and progenitor cells to specific spatial and biochemical cues.	PDMS stamp cast on starPEG–heparin hydrogels.	Grooves, rings, and cubes from 2–500 µm.	[69]
Create a bilayered hydrogel dressing to induce revascularization and re-epithelialization.	Platinum-catalyzed PDMS cast on gelatin hydrogels.	Sharklet^TM^ micropatterns of 1 µm H–10 µm W and 10 µm H–50 µm H.	[91]
Study the effects of nanotopograhical cues on hMSCs osteogenesis.	UV curable polyurethane acrylate coated with gelatin.	Nanoscale dots of 150, 400, and 600 nm diameter and lines of 150, 400, and 600 nm width.	[92]
Create dermal-epidermal regeneration matrices with microfeatures to mimic the DEJ and to study their effect on basal keratinocyte functions.	PDMS mold on stamped on collagen I—GAG gel, conjugated with fibronectin.	Micro channels with a depth of 200 µm and widths of 50, 100, 200, and 400 µm.	[93]
Investigate the effects of micro spatial cues on adipose-derived stem cells differentiation.	PDMS molds on a collagen—silk fibroin substrate.	Microchannel and micropillar patterns of 10 µm and 8 µm respectively.	[94]
To culture neonatal human fibroblasts (NHFs) to study the dermal papillae.	PDMS mold cast on Gelatin-chondroitin-6-sulfate-hyaluronic acid substrate.	Undulated microtopographies that range from 150–450 µm height and 364–1062 µm width.	[95]
Characterize the effects of topographical cues on primary human keratinocytes.	PDMS patterns coated with collagen type I.	Patterned substrates with undulations that range from 100 to 300 µm.	[96]
Study the effects of surface treatment and microgrooves on rat dermal fibroblasts.	PDMS molds treated with UV, RFGD, or a combination of both.	Square grooved surface with features of 2, 5, or 10 µm width and 0.5 µm depth.	[97]
Study the effect of surface topography on abdomen fibroblasts.	PDMS mold.	Square wells with micro topographical cues of 2, 5, or 10 µm.	[83]

**Table 3:** Summary of pattern hydrogel constructs used to recreate the micro and nano spatial cues of the stem cell niche.

Application	Polymer	Outcome	Ref
Mimic the ECM 3D structured of BMSCs to study cell-matrix interactions.	Photocrosslinked collagen hydrogel.	Porous network collagen hydrogels. Average pore size of 0.3–0.7 µm and average fiber size <100 nm.	[120]
Replicate the structural and biochemical cues of the bone marrow microenvironment in vitro.	PEG- diacrylate hydrogel loaded with relevant niche biomolecules.	Functionalized microwells of 500 μm depth and 5.34 mm in diameter.	[103,121]
Develop a platform to study how hHSC behaves when exposed to ligands expressed in their microenvironment.	PEG-diacrylate and RGD modified PEG acrylate hydrogel.	Hexagonally ordered arrays of homogeneously distributed gold nanoparticles. An interparticle distance of 40 and 90 nm.	[115]
Observe the cellular behavior of hASCs exposed to a 3D micropattern environment.	Dual-Crosslinked oxidized methacrylated alginate-PEG hydrogel using a photomask to create the micropattern.	Micro checkerboard tile patterns with dimensions of 25, 50, 100, or 200 μm.	[122]
Create an in vitro platform that mimics the native myocardial matrix of the cardiac stem cell niche.	A UV curable polyurethane acrylate mold cast on a PEG hydrogel using CFL.	An array of ridges with 400 nm width and 500 nm height, and grooves of 400 nm width.	[119]
Create a platform to study the effects of topographical cues on 3D substrates for hMSCs and hiPSCs	Alginate-gelatin and κ-carrageenan hydrogels created using micropatterned wax molds	1000 μm circular projections with 400 μm channels and 1500 μm circular projections with 600 μm channels, and square grids of 620 μm, ridges of 330 μm and channels of 270 μm.	[123]
Study the interactions of limbal epithelial stem cells inside bioengineered limbal crypts	Hydrophilic porous absorbers with microtopographies on collagen I hydrogels using RAFT^TM^	Micro ridges of equal depths and widths of 100, 150, 200, or 250 µm.	[117]

**Table 4:** Summary of microfluidic devices used to recreate the micro and nano spatial cues of the cell microenvironment.

Application	Polymer	Outcome	Ref
Create a microfabrication platform to study adult NSC fate	SU-8 photoresist material coated with poly-ornithine and laminin, placed on oxygen plasma treated glass coverslips	An array of microwells with dimensions that ranged from 20 to 500 µm in diameter and 10–500 µm in height.	[137]
Study the effects of 3D microenvironment for NSCs on self-renewal and differentiation	PDMS surface coated with COL I fabricated with a SU-8 pattern master. A COL I hydrogel was used as a cell carrier	3D collagen-coated microchannels of 140–160 μm height.	[138]
New fabrication approach to recreate stem cell niches using hydrogel engineering with droplet microfluidic technology	PDMS microfluidic bonded to glass coverslips using oxygen plasma. Chips were loaded with functionalized PEG hydrogels.	Microchannels array of 100 μm deep with three different channel widths of 100, 200, and 300 μm.	[139]
Generate a high-throughput platform to study the stem cell microenvironment with a tunable ratio of encapsulated species.	Cell-laden agarose microgels loaded into a functionalized PDMS surface.	An array of micro agarose gels of 70 to 110 µm.	[140]
Build functional networks that can be modified during the experiment to manipulate hMSC behavior in situ.	PDMS mount to cast crosslinked PED hydrogels	Artificial blood-vessel microfluidic network within cell-containing hydrogels. Channel diameter can be controlled in situ.	[141]
Create a two-layer microfluidic system to culture 3D multi-cell type spheroids to study cancer stem cell microenvironment.	PDMS device separated by a polycarbonate membrane and treated with 1% *w*/*v* Pluronic F108	A microfluidic system with a lower channel of 100 μm H and 2 mm in W, and a central microchannel of 200 μm H and 50 μm in W.	[142]

**Table 5:** Summary of electrospun constructs used to recreate the micro and nano spatial cues of the stem cell niche.

Application	Polymer	Outcome	Ref
Study adhesion and expansion of hHSCs	Polyethersulfone (PES) aminated using acrylic acid	Non-woven PES nanofiber meshes of 529 ± 114 nm in diameter.	[159]
Study the effects of fiber diameter on NSC differentiation and proliferation	Laminin-coated PES mats	Electrospun fiber meshes with average diameters of 283 ± 45 nm, 749 ± 153 nm, and 1452 ± 312 nm	[160]
Observe the sensibility of NSCs when exposed to an aligned topography	PCL fibrous mats coated with polyornithine and laminin	Aligned electrospun fibers with average diameters of 251, 472, 923 nm, and random fibers of 269, 481, 934 nm.	[161]
Study the influence of transplanting MSCs and ESCs in re-epithelization	Silk fibroin protein/gelatin polymer solution	Random or aligned uniform bead-less fibers with diameters of 63.1 ± 2.7 nm	[162]

**Table 6:** Summary of tissue engineered devices created to replicate the micro and nano spatial cues of the stem cell niche by combining two or more fabrication techniques.

Application	Polymer	Outcome	Ref
Develop fibrous membranes with controlled microenvironments to study MSC behavior	SLM metallic collectors used as templates for PCL fibers	Three different topographies were tested with dimensions 667, 1038, and 1168 µm. Average fiber diameter of 1.8–2.2 µm	[165]
Study of osteogenesis of hMSCs using sequential delivery of multiple growth factors	PCL/gelatin fibers incorporated into PEG-diacrylate hydrogels	PCL/gelatin microfibers of 1.32 ± 0.11 μm in diameter loaded into square pattern arrays of 1 × 1 mm	[179]
Design artificial limbal stem cell niches using biodegradable electrospun rings containing microfeatures	Polyethylene glycol diacrylate (PEGDA) collectors used with PLGA 50:50 fibers	Constructs of 1.2 cm diameter and 0.36 mm thickness containing U-shaped micro pockets of 150–300 µm diameter made of microfibers of ~3.5 µm in diameter.	[167]
Create patterned scaffolds to simulate the anisotropic and multiscale architecture of cardiac tissue, to promote cardiac cell alignment	Teflon-coated silicon wafer patterned collector to use with a blend of poly(glycerol sebacate) (PGS) and PCL	Fibrous constructs with an average fiber diameter of 1.2 µ and three patterns tested: Two arrays of parallel grooves of 10 μm, and square shaped features of 100 μm.	[180]
Develop a new in vitro model in which to study epithelial stem cell behavior	Poly(3-hydroxybu- tyrate-co-3-hydroxyvalerate (PHBV) fibers patterned using a PEGDA template	Fibrous bilayer constructs with an average fiber diameter of 750 nm. The micropattern layer was made of square or rectangular features of 200–1000 µm in width and 200–500 µm in depth.	[166]
Create a platform to mimic the cellular microenvironment of hMSCs	Oxygen plasma treated PDMS microfluidic device with carboxyl group modified PU fibers	Microfluidic chip with randomly orientated nanofibers of 200–500 nm diameter.	[181]
Study the use of a sandwich-type scaffold to promote re-epithelialization	Stainless steel collector coated with plasma treated PCL polymer fibers	Random and aligned fibers with microwells of 200–280 µm in depth. No fiber diameter was reported.	[182]
Develop a hybrid scaffold to study chondrogenic differentiation of hMSCs based on protein and gene expression	Composite of a thermosensitive PEG-PNIPAAm gel and PCL fibers	An electrospun scaffold of ~11 µm fiber diameter encapsulated in a mold-less hydrogel.	[183]
